# JAK-STAT Inhibitors in Atopic Dermatitis from Pathogenesis to Clinical Trials Results

**DOI:** 10.3390/microorganisms8111743

**Published:** 2020-11-06

**Authors:** Krzysztof Szalus, Magdalena Trzeciak, Roman J. Nowicki

**Affiliations:** Department of Dermatology, Venereology and Allergology, Medical University of Gdansk, 80-214 Gdańsk, Poland; kszalus@gumed.edu.pl (K.S.); roman.nowicki@gumed.edu.pl (R.J.N.)

**Keywords:** atopic dermatitis, JAK-STAT, abrocitinib, baricitinib, upadacitinib, tofacitinib, delgocitinib, ruxolitinib, cerdulatinib, gusacitinib

## Abstract

A common disease worldwide is known as atopic dermatitis (AD), named also as atopic eczema, which is a chronic recurrent complex inflammatory skin disorder. It affects 2–10% of the adult population and up to 20% of the pediatric population. The clinical AD picture appears in typically localized eczema and dry skin, and is dominated by a persistent pruritus followed by sleep disturbances. AD strongly impacts on the quality of life of AD patients and their families as well as on social and economic aspects. The pathogenesis of the disease is complex and consists of multiple interactions between immunological disturbances, skin barrier defect, and microbial dysbiosis with environmental influences. The treatment of AD reflects the pathogenetic disorders, starting from basic emollient therapy, and goes to topical anti-inflammatory regimens followed by phototherapy, systemic immunosuppressive drugs, and new biologic immunomodulators. This paper will thus summarize the novel collection of biological treatment JAK-STAT inhibitors dedicated to AD.

## 1. Introduction

**Figure 1:**
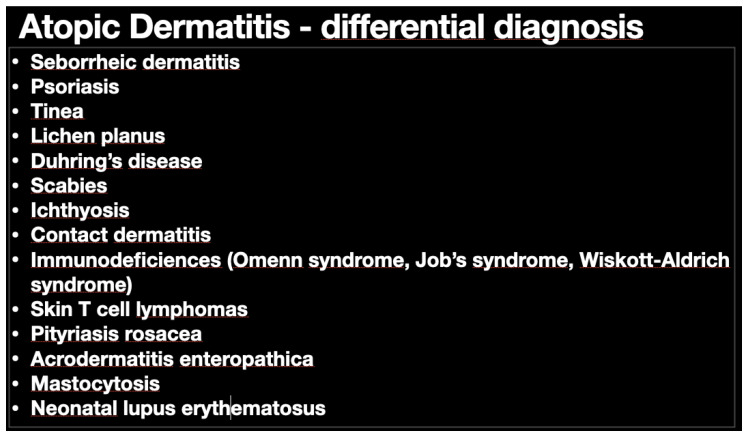
Differential diagnosis of atopic dermatitis (AD). Main potential diseases that can confuse AD diagnosis are included in the table. Many different options do make this an easy way to reach final diagnosis.

**Figure 2:**
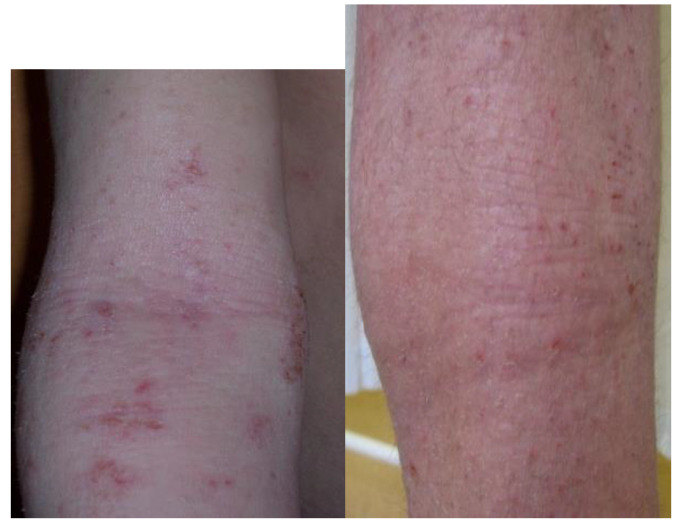
Typical location and morphology of atopic eczema. Photos show typical location of skin lesions in children.

Atopic dermatitis (AD) as an inflammatory disease that mostly affects children and less adults. The prevalence of this is still rising and affects between 10% and 20% of children in the global population, starting in early childhood. The majority of symptoms appear before the first year of life in about 60% of children. Another 30% with AD will exhibit their first symptoms between their first and fifth year of life and the minority (10%) after their sixth year of life [1]. Adult population with AD prevalence oscillates between 2.1% to 4.9% globally [2]. There is no specific markers to indicate while diagnosing AD, so the long, sometimes troublesome diagnostic process is based on clinical symptoms following the criteria of Hanifin and Rajka [3] and medical records of the patient after exclusion of other inflammatory mediated diseases (Figure 1). Clinical picture beside pruritus and dry skin manifests as exematouse lesions localized mainly in flexures, but it may spread through the whole body. In infants, thee extensor side is typical (Figure 2). The treatment of AD reflects the pathogenetic disorders. Current management of AD is a combination of basic emollient therapy goes to topical anti-inflammatory regimens followed by phototherapy and in the case the other treatment fails, moving to systemic immunosuppressive drugs. Although many schemes depend on the age and severity of the disease, we are still lacking success in the treatment of moderate and severe AD. The need for novel treatments with a steroid sparing effect, to encompass the potential side effects of topical corticosteroids over the long-term use is crucial. Thus, there is a need to search for better solutions of treatment, control, healing, and prevent the disease and its consequences like so called “atopic march”. Better understanding of AD pathogenesis, its immunological mechanisms, a variety of monoclonal antibodies targeting cytokines, and small molecules interfering with intracellular signaling pathways have been developed and let us create novel drugs that do not focus on a single pathological pathway of the disease, but more complex molecular interactions that drive AD. We also observed new possible and effective trends of treating atopic dermatitis mostly by focusing on a personal conception of the patient’s disease like AD immuno/geno/phenotypes.

## 2. Pathogenesis of Atopic Dermatitis 

**Figure 3:**
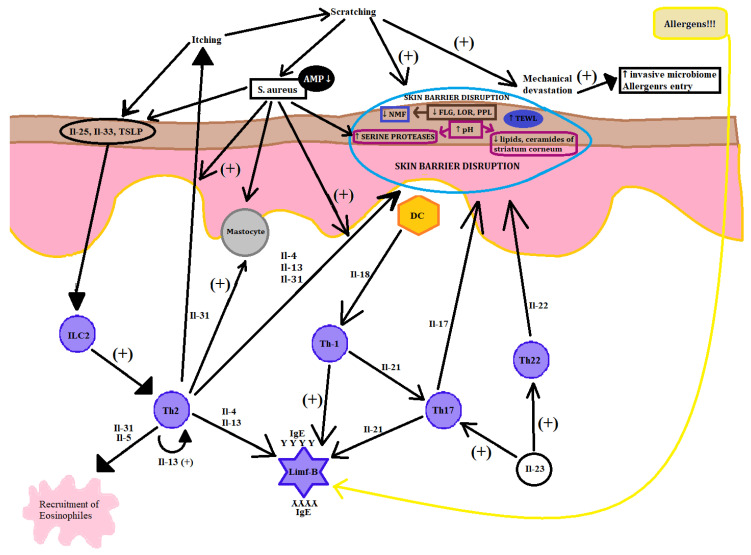
AD pathogenesis. A compound process in the picture unveiled the combined pathogenesis of AD that leads to the acute phase of the disease. The picture is simplified to understand the influence of many different factors including environment on AD focus. Key (+: stimulation, ↑: increase, ↓: decrease).

AD pathogenesis is complex (Figure 3) and includes disorders in skin barrier, immunology, and skin microbiota. AD development also depends on environmental and personal factors. 

### 2.1. Skin Barrier Defect

Molecular defects, which take place within the dermal layers manifest themselves as dysregulation of cornified envelope (CE) proteins, stratum corneum lipids, tight junctions, and overreaction of serine protease [4], explain many AD symptoms. Filaggrin (FLG) is the best searched cornified envelope protein in AD pathogenesis and when it acts properly, it is a substrate for natural moistening factor. It is obvious that the lack of FLG results in skin dryness, but not only as filaggrin mutations are associated with severe AD course, concomitant asthma, and elevated IgE level [5]. Further consequence of FLG disorder is incorrect skin pH, which creates unfavorable conditions for lipid synthesizing enzymes. The decreased expression of FLG and the CE proteins is the effect of gene mutations as well as chronic inflammation in the atopic skin [6]. Aside from different expressions of cornified envelope proteins like FLG, lorikrin (LOR), cornulin, (CRNN), repetin (RPTN) and small proline-rich proteins (SPRRs) [7], the stratum corneum is deprived of intracellular lipids like ceramides [8]. One of the mechanisms is connected with the maturing of lamellar bodies [9]. The skin barrier defect is recognized in normal skin without the lesions and within lesionally changed skin regions in AD patients [10]. Epidermal barrier dysfunction results in increased permeability, reduced cohesia, and integrity of the epidermis, increased transepidermal water loss (TEWL), drying of the skin, and tears and ruptures of the skin [11]. Epidermal barrier defect facilitates penetration of allergens, leading to epidermal sensitization and allergy development [12]. The anti-inflammatory treatment indirectly facilitates restoring the skin barrier defect by diminished inflammation, which can secondarily deepen the skin barrier defect [13].

### 2.2. Microbiota in Atopic Dermatitis (AD)

AD dysbiosis and poor physiological microflora diversity is a well-known condition in AD. It is known that more virulent strains of *Staphylococcus aureus* are predominant in AD exacerbation and is associated with AD severity [14]. Regular non-lesional skin is colonized by a commensal type of bacteria as well as *S. aureus*, which contributes to the maintenance of skin defensive factors and protection against invasive microbiota [15]. Lack of antimicrobial peptides in AD and disorders of innate immunity including Toll like receptors favor virulent strains of *S. aureus* contamination [16]. Thanks to different mechanisms, *S. aureus* is able to interfere with the skin barrier. With its adhesion particles, clumping factor A and B, fibronectin-binding protein, and iron-regulated surface determinant A, *S. aureus* is able to adhere to the human skin. Moreover, it creates heptameric β-barrel pores in keratinocytes and cell membranes that destroy the unity of the epidermal skin barrier as well as the secretion of proteases to dissolve stratum corneum. *S. aureus* induces the inflammatory process via staphylococcal super-antigens like SEA, SEB, SEC, and toxic shock syndrome toxin-1 (TSST 1), which triggers cytokine release and influx of leukocytes [17]. *S. aureus* is involved in promoting the inflammation process in AD skin and deepening the skin barrier defect. The *S. aureus* virulence mechanism includes the activity of enterotoxins and alfa delta toxins, proteases that through Th-lymphocytes, mast cells, DCs, and IL-31 increase the ice sensations and by IL36, Il17, TSLP, and Th2 cytokines promote inflammation [18]. There is medical research investigating the impact of Th2 lymphocyte inflammation on the skin microbiota in patients with AD. Such research says that targeting the Th2 lymphocyte way of inflammation with drugs like dupilumab may improve diversification of microbiota and reduce colonization lesional and non-lesional skin with *S. aureus* and may have potential impact on the modification of the disease. Moreover, control mechanisms of atopic march with dupilaumab usage are still under consideration [19]. There is an open question of if and how the JAK_STAT inhibitors influence AD microbiota.

### 2.3. Immunologic Disorders

Immunologic disorders in AD consist of innate and adaptive immune response disorders. The major AD pathological pathway is based on the Th2 lymphocyte axe activation of the inflammatory process. Although Th17, Th22, and Th1 cytokines are also involved depending on AD phase, patient age, and ethnic background [20,21,22,23,24]. Various things as mechanical injuries, allergens, and invasive microbiota can trigger and accelerate immune mechanisms of skin, causing rapid response of increasing the expression of IL-25, and IL-33 in the skin innate immune system, further activating the cascade of Th-2 lymphocyte response. Then, interleukins 4,13,22 amplify the Th2 lymphocyte response and downregulate the cornified envelope proteins (FLG, LOR, PPL, and claudins expression) as well as inhibit the expression of defensive epithelial barrier proteins and terminal differentiation of Keratinocytes. Th2 lymphocytes are also responsible for the production of IL-31, so called “pruritis cytokine”, that are found in large amounts in skin acute lesions, which takes part in the itch–scratch cycle along with other mediators like histamine, tryptase, and neuropeptides. Moreover, Th2 lymphocytes also contribute to the secretion of IL-5 that promotes an influx of eosinophils and propagation of the inflammatory process. It is considered that the acute phase of the disease is strongly modulated by Th2 and Th22 lymphocytes, but modern investigations have discovered the huge impact of Th17 lymphocytes and IL-17 and IL-23, which modulate the pathology of the acute phase of AD. Th17 lymphocytes are known to be fundamental mediators of psoriasis by the production of IL-17, however, IL-17 contributes to maintain the inflammation process in AD and is the chemokine for neutrophils and T lymphocytes. Some of the newest clinical trials indicate that Th17 lymphocytes may impact on the propagation of IL-4 in AD. Thus, the AD acute phase is mostly generated by the activation of Th2 and Th22 lymphocytes, and the chronic lesions show the impact of Th1 lymphocyte component activity. Activation of the Th1 lymphocyte pathway connects to upregulation for interferon (IFN) gamma and IL-12, which promotes the chronic phase of inflammation and the Keratinocyte apoptosis process [20]. Although the Th2 lymphocyte axe is universal for the majority, there are still many other cytokines involved into triggering the disease. AD can be classified as an intrinsic and extrinsic. The majority (80%) presents the extrinsic type of AD. The difference lies in the IgE serum level and only the extrinsic type expresses a high level of that cytokine as well as positive medical records for allergies and atopic diseases in family. The research performed on (51) patients divided into extrinsic (42) and intrinsic (9) groups unveiled stronger activation of all inflammatory pathways based on the Th2 lymphocyte axe, especially including particular Th17 and Th22 lymphocyte cytokine pathways in the intrinsic type of AD with normal serum level of IgE and negative family allergy history. Only extrinsic AD patients expressed correlation between SCORAD scores and high IgE serum level and skewing Th17 and Th22 lymphocyte cytokine levels with preserved Th2 lymphocyte activation [21]. In Asia, there is a slight difference in immunological pathomechanism. Recent survey has shown the AD phenotype among the Asian population is between European–American and the psoriatic one. This is caused by the higher influx of Th17 and Th22 lymphocytes as well as the high level of IL-17 and IL-19 in Asian AD in lesional skin, high level of IL-22 in nonlesional skin, and low interference of Th1 and low level of interferon gamma. This molecular difference manifests as a greater acanthosis and more frequent paraceratosis in Asian AD patients. Additionally, for this group of patients, there was a high IgE serum level diagnosed [22]. For European–American AD type, there is a common knowledge regarding interleukins of type 2 inflammation, especially IL-4 and IL-13, as responsible factors for the disease, causing epithelial barrier dysfunction, allergic inflammation, microbiota dysregulation, and natural dermal antimicrobial peptide dysfunction. In the acute phase, studies observed that the growth of expression of TSLP, IL-25, and IL-33 in the skin and production of those cytokines was triggered by allergen entry, invasive microbiota proliferation, cigarette smoke, and other chemical irritants. These immunological factors subsequently effect dendritic cells and lead to immunological reaction via influx of Th2 mostly, and also Th22 and Th17 lymphocytes that were most prominent in Asian atopic dermatitis patients. The activation of these lymphocytes provides upregulation of IL-17, which reduces the level of FLG, IL-22, which is responsible for skin barrier dysregulation; IL-13, which strengthens the whole process; and IL-31, which gives the feel of the itch [23]. When it starts the transition to the chronic phase, the role of the activation of Th1 lymphocytes becomes significant and is followed by the growth in the interferon gamma level with sustained activation of lymphocytes Th2 and Th22 [25]. There is also the difference between the immunological implications in the skin of children and adults. Both types have a huge impact in the activation of the Th2 axe, but there is a significant relation of IL-17 with inflammation in AD early onset in the pediatric population. Moreover, the skin of adults unveiled an increase of Th22 lymphocyte polarization, which probably correlates with constant immuno-stimulation over time [24]. From this perspective, AD seems to have different subtypes built on molecular based immunotypes/genotypes [26]. Knowing the immunotype of the AD patients, therapeutic plans may be more precise in treatment and more effective with new biological agents like dupilumab (anti IL4R monoclonal antibody) and more like new biological antibodies blocking the IL-13, IL-22, and IL-17. Dividing patients into intrinsic and extrinsic types will help to provide the appropriate treatment. This means that we may use personalized medicine with monoclonal antibodies against particular cytokines or their receptors. Such a strategy is useful for increasing the efficacy of the treatment. However, currently, we may also use agents directly against groups of cytokines to influence JAK-STAT signaling or phosphodiesterase inhibitors (PDE) [27]. Moreover, there are other components that have an effect on the immunology system like vitamin D, which is known to have a pleiotropic effect. For example, calcitriol, which is an active form of vitamin D and is known to suppress STAT 1 and STAT 3 phosphorylation, has been proven in an autoimmune disease mouse model [28]. However, the role of vitamin D in AD is still being discussed and further studies amongst AD patients are necessary to reach conclusions. These all show how compound is the pathogenesis of AD. Another scientific study showed that there were complex relationships between AD, vitamin D, and body mass index (BMI). The study was conducted among a pediatric population with AD and without AD living in cities. Evaluation concerned Scoring Atopic dermatitis (SCORAD), BMI, and vitamin D level in serum. There was a correlation between vitamin D level and occurrence of AD, and another one between BMI and severity of AD in males. The outcome exposes the compound relationships of vitamin D, AD severity, and BMI and enhances the need to study many factors such as vitamin D level and BMI and genetic predispositions to better understand and develop new strategies [29].

## 3. JAK STAT Signaling

**Figure 4:**
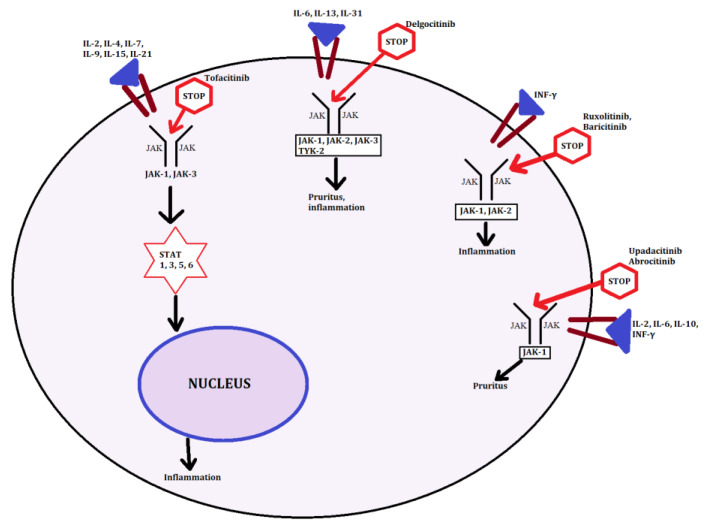
JAK-STAT targeting sites. In the figure, we present grip points followed by the particular JAK-STAT pathway for major inhibitors described in the article.

The JAK STAT pathway is activated by the key mediator IL-4. Activation of JAK is followed by phosphorylation of STAT, which in its active state translocates to the cell nucleus to target particular genes. We distinguished the JAK family including JAK1, JAK2, JAK3, and TYK2 as well as STAT family: STAT1, STAT2, STAT3, STAT5A/B, and STAT6. JAK-STAT activation has effects on the regulation of Th2 differentiation, and because of that, plays a significant role in AD. We know widely that the activation of Th1 lymphocytes of the chronic phase are responsible for JAK2, TYK2, and downstream of STAT1/4 phosphorylation mediated by the IL-12R pathway, and ultimately interferon gamma signaling [30]. However, for acute phase lymphocyte, Th2 activation is mostly responsible for JAK1, JAK3, and the next subsequent activation of STAT6 by signaling of the IL-4 pathway as well as STAT5A/B, which helps STAT6 to bind to its targets, leading to transcriptional regulation of the GATA3 target gene (Figure 4). Furthermore, STAT6 is also involved in B cell differentiation, IgE class switching, and MHC class II production, therefore many polymorphism of STAT 6 is associated with predisposition to IgE class production and allergic diseases. Loss of function mutations of TYK2 are followed by impairment of the signaling pathways of IL-1, IL-12, and type 1 interferon, leading to dysregulation of differentiation of the Th1 lymphocyte, which is correlated with susceptibility to AD, skin infections, and high IgE serum levels, so that we know TYK2 plays an important role in Th2 lymphocyte differentiation. STAT3 is one of the agents responsible for the expression of IL-23 triggered by IL-6 secreted from DC cells that is vital for Th17 lymphocyte differentiation and critical for cell memory, resulting in destroying the epithelial barrier integration [31]. Thus, upregulation of Th2 immune response by the JAK-STAT pathophysiology pathway is followed by hyper secretion of different cytokines, angiogenic factors, chemokines for eosinophils, and growth of IgE level that binds to mast cell receptors and exacerbate the AD inflammation process. Described above, the increased Th2 immunity driven by JAK-STAT signaling downstream of cytokines such as Il-4, Il-5, and Il-13 is a very important pathogenic pathway of AD [32]. Studies have shown the JAK-STAT pathways are involved in mediating the inflammation process and changes of natural skin barriers, and increased TEWL by interactions of a number of cytokines causing AD by stimulating the expression of IFN-γ, IL-31, IL-23, and IL-22. Several topical and oral JAK inhibitors have been shown to decrease AD severity and symptoms [33]. Nowadays, the JAK inhibitors are undergoing an evaluation of safety and efficacy in the treatment of AD. Moreover, other alterations such as interactions with other drugs are being conducted. The results seem to be promising in finding a new class of medications. We performed the juxtaposition of several JAK inhibitors to show the complex molecular nature of AD and the different drug grip points in AD.

## 4. JAK Inhibitors in Atopic Dermatitis (AD)

There are several JAK inhibitors of the first generation: tofacitinib, ruxolitinib, and baricitinib as well as newer ones like upadacitinib, abrocitinib, cerdulatinib, gusacitinib, and delgocitinib. Food and Drug Adminisration (FDA) approved tofacitinib was the first one to cure autoimmune diseases like rheumatoid arthritis, then psoriasis, and is now undergoing clinical trials in AD. Indication of FDA for ruxolitinib is myelodisplastic disorders, and baricitinib is in clinical trial for RA—phase 3, psoriasis, and AD—phase 2, and is not yet FDA approved for anything. Moreover, we have a wide spectrum of drugs with particular action inhibiting single transcriptors as filgotinib-JAK1; pacritinib-JAK2; decernotinib-AK3, and nonselective drugs that inhibit more than one (e.g., tofacitinib-JAK1/2; ruxolitinib and baricitinib-JAK1/2) [34].

Abrocitinib is an oral selective inhibitor of JAK 1 being investigated for efficacy and safety profile in the treatment of patients with signs and symptoms of moderate to severe AD. The drug is being tested in a phase 2b, randomized, doubled-blinded, placebo-controlled clinical trial including 267 adult participants with at least one year of AD and with inadequate response or contraindication to topical treatment for four weeks or more within the 12 months. The results of taking this 12 week clinical trial of once daily oral dose of 200 mg, 100 mg, 30 mg, 10 mg abrocitinib were mostly the reduction in Eczema Area and Severity Index (EASI) score by 82.6% for those receiving 200 mg, 59% for those receiving 100 mg of abrocitinib, and 35.2% placebo effect as well as improvement in the Investigator Global Assessment (IGA) from baseline at week 12. The most common side effects occurred in 184 out of 267 participants. The most frequent side effects were upper respiratory tract infection, headache, nausea, and diarrhea. Researchers also reported a dose-dependent decrease in platelet count from baseline, but after week 4, it went up to the baseline. There were also various limitations such as a small sample size and short period of the treatment. In phase 3 of this trial, the dose of 100 mg and 200 mg of abrocitinib will be evaluated more precisely for safety and efficacy [35]. The new study JADE-MONO-1 showed that abrocitinib succeeded in effectiveness and tolerance in adults and adolescents with moderate to severe AD. Patients were assessed for 12 week efficacy. IGA response was higher in the 100 mg abrocitinib and 200 mg abrocitinib group than in the placebo. Patients who achieved EASI 75 showed a response that was higher in the 100 mg abrocitinib group and abrocitinib 200 mg group. There were no deaths reported. Adverse effects were reported in both the 100 mg and 200 mg abrocitinib groups [36] (Table 1).

Baricitinib is a selective JAK1 and JAK2 inhibitor that regulates the pro-inflammatory cytokine signaling pathway with no specific FDA indications. The research was performed on 124 adult patients with moderate to severe AD. The phase 2 study was randomized, double-blind, placebo-controlled, and was designed to reduce EASI score compared to the placebo. Each patient was administrated with topical corticosteroids (TCSs) for four weeks prior to the start of the experiment and was also allowed to use the TCSs during the experiment. Study has shown a significant reduction by 50% in EASI scale among patients taking an oral dose of baricitinib 4 mg and 2 mg vs. patients who received the placebo in a 16 week trial. Already in the first week, there was an improvement in the EASI scale. During the trial, there was a reduction in pruritus as early as the first week and by week 4, pruritus was decreased by as much as 45% among patients taking baricitinib 4 mg daily vs. the placebo. In addition, baricitinib reduced sleep loss and decreased the SCORAD level. Overall, general improvement in the Dermatology Life Quality Index (DLQI) was observed for both 4 mg and 2 mg doses of baricitinib at week 4. Side effects were reported in 27 of those receiving 4 mg of baricitinib, in 24 of those receiving the placebo, and in 17 patients receiving 2 mg of baricitinib. The reported side effects were mostly headaches, rhynopharyngitis, increase of CPK, decrease of WBC, neutropenia, lymphopenia, and exacerbations of AD observed in ones on the placebo and a colonic polyp was reported in the large intestine of one patient at 4 mg of baricitinib. It seems necessary to provide further and longer studies on baricitinib’s efficacy and safety in AD because TCSs have been used prior to randomization, reducing the severity of AD [37]. The new study from August 2020 of BREEZE-AD1 and AD2 revealed that baricitinib efficiently reduced the severity of AD in phase 2 in patients with inadequate response o topical corticosteroids. Baricitinib also affected rapid itch response and the drug was well tolerated. Results were collected at 16 weeks. Validated Investigator’s Global Assessment was achieved for groups who received baricitinib 4 mg and 2 mg compared with the placebo in BREEZE-AD1 and BREEZE-AD2. For pruritus the improvement was reported in first week for 4 mg dose and in second week for 2 mg dose of baricitinib. Therefore, improvement of skin pain, sleep, and quality of life indicators was observed in the first week of treatment at both thee 4 mg and 2 mg dose. Nasopharyngitis and headache were mostly reported as adverse effects [38] (Table 1).

Upadacitinib is a selective JAK1 inhibitor. Study on upadacitinib on pruritus was conducted on adults with moderate to severe AD in a randomized, double-blind portion phase 2b 88-week trial. The results showed the significant improvement in pruritus not a day before day 2 [39]. Now phase 3 is currently underway, comparing the safety and efficacy of upadacitinib to dupilumab in adult with moderate to severe AD. Another clinical trial of phase 2b on upadacitinib was performed to investigate the safety and efficacy of multiple doses in patients with moderate to severe AD. This was a double-blind, randomized, parallel group trial on 167 patients and 166 received upadacitinib or a placebo for 16 weeks. Results of efficacy were reported from a 16 week period, all upadacitinib doses (7.5 mg, 15 mg, and 30 mg) showed statistically significant mean percentage improvement from the baseline in EASI vs. placebo (39% *p* = 0.03, 62% *p* < 0.01, 74% *p* < 0.01 vs. 23%, respectively). For the IGA of 4 (severe AD) the improvement from baseline in percentage was 35% *p* = 0.19; 59% *p* < 0.01, and 75% *p* < 0.01 versus 19% for placebo control in the upadacitinib 7.5-, 15-, and 30 mg groups, respectively. In the survey, the achievement of EASI 100 was done by 2.4%; 9.5% and 24% vs. 0% placebo of groups of patients taking upadacitinib 7.5-, 15-, and 30 mg, respectively. There was also important improvement in itching as assessed in Numerical Rating Scale (NRS) by a NRS reduction equal and/or greater than 4 at week 16. Efficacy of upadacitinib was observed by weeks 1 and 4. Biopsies from lesional and non lesional skin areas from 50 patients showed a reduction of epidermal hyperplasia and number of dendritic cells in upadacitinib 15 mg and 30 mg. Side effects were reported in 71%, 74%, and 79%, respectively for upadacitiinib 7.5 mg, 15 mg, and 30 mg versus 63% placebo. The most common reported side effects were upper respiratory tract infections, AD worsening, and acne with no correlation of dose of the drug with particular adverse effect. Then a new study on upadacitinib reported a dose-response relationship. Dose of 30 mg once daily turned out to be the most effective with the best clinical benefits at 16 week of treatment [40] (Table 1).

Tofacitinib is the first of the novel drugs and is a nonselective JAK inhibitor, with stronger prevalence to JAK1/3 with proven results published of phase 2 trials. Tofacitinib inhibits activation of JAK1/3 via their phosphorylation and subsequently stops binding to their receptors with blockage of STATs-1/3/5/6, thereupon they cannot translocate to the cell nucleus and trigger the transcription of pro-inflammatory cytokines. Tofacitinib has a minimal effect on TYK2 and JAK2 [41]. In the spectrum of tofacitinib activity are DCs, T cells CD4+ like Th1 and Th17, and activated B-cells [42]. The FDA strongly recommends it for rheumatoid arthritis at a 5 mg dose twice daily and it inhibits in experimental mouse models of AD Il-4 and Il-13. In six cases of adults with moderate to severe AD who have failed conventional therapy, tofacitinib was reported as an effective systemic drug. Tofacitinib was administrated orally 5 mg twice daily or 10 mg daily during eight to 29 weeks. The result was a reduction by 66.6% in the SCORAD during eight to 29 weeks, a reduction in pruritus by 69.9% of cases, and also decreased the area of dermatitis on the body surface with a drop in the erythema, edema, and lichenification in all patients. No side effects were reported in the study. This research indicates the promising utilization of the use of tofacitinib in AD, in which common systemic treatment fails [43]. However, there is much to prove in the efficacy of a JAK inhibitor in autoimmune skin disorders. Another clinical phase 2a, randomized, double-blinded trial with 2% tofacitinib ointment was performed on 69 adults with mild to moderate AD for four weeks. Patients were randomized 1:1 to a 2% tofacitinib ointment and vehicle ointment twice daily. The EASI score was reduced by 81.7% for tofacitinib vs. 29.9% for the placebo. Important improvements in EASI, Physician’s Global Assessment (PGA), and body surface area (BSA) were observed by week 1, moreover, significant improvement in pruritus was obtained by day 2. The interesting fact was that the adverse effects were observed more for the vehicle vs. tofacitinib and the general toleration was similar for both medications [44] (Table 1).

Delgocitinib has an inhibiting effect on all JAK kinases 1,2,3 as well as TYK2. In an animal AD model, delgocitinib has been shown to be an effective factor in suppressing skin inflammation with topical use. A phase 2 randomized, vehicle controlled clinical trial was conducted on 327 Japanese adult participants with moderate to severe AD to evaluate the efficacy and safety profile of the delgocitinib ointment. The outcome of that study with 309 adults revealed a decrease in EASI score by 41.7%, 57.1%, 54.9%, 72.9%, and 12.2%, respectively for 0.25%, 0.5%, 1%, 3% twice daily and the vehicle over four weeks. Pruritus decreased in NRS score at day 1 at night. Delgocitinib doses up to 3% were well tolerated with only mild to moderate side effects and a drop in WBC [45]. Another similar study was performed on a pediatric population. This phase 2 clinal trial was randomized, double-blind, vehicle controlled, and conducted on patients aged two through to 15 years with AD. They received 0.25% and 0.5% ointment vs. placebo twice daily applied on the inflamed area and eczema for four weeks. A total of 103 participants started the study and 98 finished the study. Five participants from the vehicle group did not complete the study. The outcome for delgocitinib revealed that it had a significant effect on the reduction of EASI by 54.2% for 0.25% ointment and by 61.8% for 0.5% ointment vs. 4.8% for the placebo. Delgocitinib also had a statistically significant improvement on IGA score and pruritus compared to the placebo. Some of the side effects were reported in group of patients taking delgocitinib: 26 out of 68 cases and 17 out of 35 treated with the vehicle, but no serious side effects were reported [46]. The new study from 2020 on delgocitinib ointment was aimed at the assessment of its efficacy and tolerance. It was performed only on Japanese patients with a vehicle controlled period of four weeks. The results showed an improvement in modified EASI score in the delgocitinib group. Adverse effects were mild and uncorrelated with delgocitinib [47] (Table 1).

Ruxolitinib (RUX) is a selective Janus kinase 1 and Janus kinase 2 inhibitor that is likely to strongly suppress cytokine signaling in the AD pathogenic pathway and is indicated in myelofibrosis and polycythemia vera in 5–25 mg twice daily in both cases by the FDA. The studies on the effect of ruxolitinib on AD concerned about its topical use as a cream (CR) in phase 2 double-blind, randomized therapy. The research on the topical use of ruxolitinib 1.5% cream taken twice daily, 1.5% taken once daily, 0.5% taken once daily, and 0.15% taken once daily for eight week long treatment and vehicle or triamcinolone cream 0.1% for four weeks, and then for another four weeks with only the vehicle from triamcinolone. After that time, patients were allowed to apply topical 1.5% RUX cream twice daily. A study was performed on 307 adults with mild to moderate AD, aged between 18 to 70 years and 3% to 20% affected body surface. The IGA baseline was 2 to 3 points. The end point of the trial was by the week 4 comparison of change in EASI score from the baseline for the 1.5% RUX CR and vehicle CR. This result showed the therapeutical benefits of the use of all doses of RUX cream at week 4. Ruxolitinib 1.5% cream twice daily reduced the EASI score by 71.6% vs. the 15.5% placebo, RUX 1.5%, 0.5% and 0.15% once daily reduced EASI by 67%, 52.2%, and 45.4% vs. 15.5% placebo, respectively. Result for triamcinolone 0.1% cr. was 59.8% at week 4. Achievement of IGA responses was significant for more patients treated with 1.5% RUX (38%) cr. twice daily vs. vehicle (7.7%) and not significant vs. triamcinolone cr. (25.5%) at week 4. There was a rapid reduction of the pruritus within 36 h with its withstanding through 12 weeks for 1.5% twice daily RUX in NRS. Moreover side effects in the clinical trial for RUX were mild to moderate and the pain in place of application was the most common one that was also reported for the vehicle. Overall, ruxolitinib cream was generally well tolerated [48]. Summing up this study of ruxolitinib cream, we know that we can obtain quick and persistent recovery of AD symptoms including a decrease in pruritus with generally well tolerated treatment. There is also another clinical trial in progress that is being performed on ruxolitinib to examine the safety, tolerance, and pharmacokinetics of RUX cream applied to the pediatric population including 2 year old to 17 year old children inclusive with AD defined by the criteria of Hanifin and Rajka. The study is in phase 1 and consists of 60 participants and is a non-randomized, parallel assignment treatment (NCT03257644) (Table 1).

Cerdulatinib is a selective topical inhibitor of the JAK and spleen tyrosine kinase (SYK) pathways. It is currently in a phase 2 randomized clinical trial. Phase 1b was performed on eight participants with AD for a two week period of time and revealed significant improvement in reversal epidermal hyperplasia, hyperkeratosis, and the reduction of inflammatory cell infiltration related to the reduction in inflammatory gene expression by the use of 0.4% topical cerdulatinib gel. The EASI was reduced by 65%. The side effects were grade 1 or grade 2 [49] (Table 1).

Gusacitinib is another oral dual selective inhibitor of JAK/SYK kinase and is now undergoing phase 1b of a randomized clinical trial. The research was planned to evaluate the systemic effect and safety profile of oral gusacitinib doses once daily at 20 mg, 40 mg, and 80 mg versus the placebo during a four week trial on 36 participants with moderate to severe signs and symptoms of AD. Gusacitinib had an improvement on reducing the EASI in comparison to the placebo. EASI 75 was decreased by 0%, 71%, and 33%, respectively, at the dose of 20 mg, 40 mg, and 80 mg compared to the 22% placebo. The outcome for pruritus was evaluated by the NRS scale −1.3 ± 2.1, −3.1 ±  2.7, −4.7 ± 2.1, respectively, for the dose of 20 mg, 40 mg, and 80 mg of gusacitinib vs. −1.6 ± 1.8 placebo. Reported adverse effects were mild and similar to all groups, and the most common was headache, nausea, diarrhea, rhynopharyngitis, back-pain, mild hypertension, and lowering the levels of lymphocytes. Gusacitinib is associated with reducing systemic inflammation [50] (Table 1).

## 5. Discussion

New evidence for the utilization of novel biological therapies targeting the JAK-STAT pathway for the treatment of AD is now being developed where the results seem promising. Regarding the complex nature of AD with different endotypes regarding immunology and skin barrier defect, it seems that the disease requires personalized solutions of treatment. Aside from monoclonal antibodies targeting one aim, drugs with a wider mechanism of action like JAK inhibitors seem to open new possibilities and hold a special place in the available drug pyramid of AD treatment. However, it also raises some points for further discussion and studies. Oral administration with good bioavailability and topical route of treatment considering JAK agonists and antagonists appear to be an advantage. Patients treated with JAK inhibitors should be monitored closely during therapy for any signs of infection. The possible synergistic effect with other immunomodulating agents also targeting the JAK-STAT pathway (e.g., vitamin D) should be considered. However, to be defined is whether selective JAK 1 inhibitors vs. nonselective JAK inhibitors would provide a better safety profile. There is still a huge need for more studies proving the long-term safety profile of listed drugs to be applied to AD treatment. The question about the influences of JAK-STAT inhibitors on microbial activity of AD is still open.

## Figures and Tables

**Table 1:** Comparison of JAK STAT inhibitors.

JAK-STAT Inhibitor	Target	Type of Application	Phase of Studies	Most Common Side Effects	Dose Related Effect	Tested Dose and Effect on AD
Abrocitinib	Selective JAK1 [36]	Oral	3	Upper respiratory tract infections, headache, nausea, diarrhea	Decrease in platelet count	For 100 mg and 200 mg—achievement EASI-75 and IGA response in week 12.
Baricitinib	Selective JAK1 and JAK2 [37,38]	Oral	3	Nasopharyngitis, headache	None	Improvement in itch was achieved in 1st week for 4 mg and in 2nd for 2 mg, improvements in night-time awakenings, skin pain obtained by 1 st week for both doses.
Upadacitinib	Selective JAK1 [40]	Oral	3	Upper respiratory tract infections	30 mg once daily turned out to be the most effective	Improvement in EASI for doses 7.5 mg, 15 mg, and 30 mg.
Tofacitinib	Nonselective JAK1/3, TYK2, JAK2 [43,44]	Oral and topical	2	No side effects in this phase were observed	None	Improvement in EASI by 1st week for 2% ointment and significant improvement in itch by day two. For 5 mg and 10 mg twice daily p.o. observed reduction of SCORAD by 66.6% and also reduction of pruritus.
Delgocitinib	Nonselective JAK1, JAK2, JAK3, TYK2, [46,47]	Topical	3	Mild white blood cells drop, headache	None	Improvement in EASI for 0.5% ointment.
Ruxolitinib	Selective JAK1 and JAK2 [48]	Topical	2	Pain in the place of application	1.5% cream was the most effective	1.5% RUX cream achieved the greatest improvement in EASI and in IGA. Also rapid itch reduction was obtained.
Cerdulatininb	Selective JAK1, JAK2, JAK3, TYK2, SYK [49]	Topical	2	Diarrhea, neutropenia,	None	0.4% gel has significant EASI improvement. Rapid pruritus NRS score reduction.
Gusacitinib	Selective JAK1, JAK2, JAK3, TYK2, SYK [50]	Oral	1	Headache, nausea, diarrhea, rhino-pharyngitis, back-pain, mild hypertension, decrease in lymphocyte	None	All doses 20 mg, 40 mg and 80 mg were superior to placebo achieving improvement in EASI 50 and 75 with highest efficacy for 40 mg
